# RCDI/eRCDI: a web-server to estimate codon usage deoptimization

**DOI:** 10.1186/1756-0500-3-87

**Published:** 2010-03-31

**Authors:** Pere Puigbò, Lluís Aragonès, Santiago Garcia-Vallvé

**Affiliations:** 1 National Center for Biotechnology Information, National Library of Medicine, National Institutes of Health, Bethesda, MD 20894, USA; 2 Department of Microbiology, University of Barcelona, 08028 Barcelona, Spain; 3 Department of Biochemistry and Biotechnology, Nutrigenomics Research Group, Rovira i Virgili University, 43007 Tarragona, Spain

## Abstract

**Background:**

The Relative Codon Deoptimization Index (RCDI) was developed by Mueller et al. (2006) as measure of codon deoptimization by comparing how similar is the codon usage of a gene and the codon usage of a reference genome.

**Findings:**

RCDI/eRCDI is a web application server that calculates the Relative Codon Deoptimization Index and a new expected value for the RCDI (eRCDI). The RCDI is used to estimate the similarity of the codon frequencies of a specific gene in comparison to a given reference genome. The eRCDI is determined by generating random sequences with similar G+C and amino acid composition to the input sequences and may be used as an indicator of the significance of the RCDI values. RCDI/eRCDI is freely available at http://genomes.urv.cat/CAIcal/RCDI.

**Conclusions:**

This web server will be a useful tool for genome analysis, to understand host-virus phylogenetic relationships or to infer the potential host range of a virus and its replication strategy, as well as in experimental virology to ease the step of gene design for heterologous protein expression.

## Findings

Some studies suggest that viral genomes may acquire replicative fitness by selection in one of two ways. First, a codon usage optimized genome, as observed for viruses with high replication rates, such as Poliovirus [1,2]. Alternatively, selection might favour codon usage deoptimized genomes, as observed for viruses with low replication rates, such as Papillomaviruses [3,4]. However, some viruses may have both codon optimized and codon deoptimized genes which may be associated with a lytic or lysogenic phase respectively [5]. In addition, some authors propose that suboptimal codons may be used as a complementary strategy to develop polio vaccines [6-8].

The Relative Codon Deoptimization Index (RCDI) was developed by Mueller et al. (2006) as a measure of codon deoptimization, by comparing the similarity in codon usage of a given gene to reference genome. Though the RCDI is a useful index for virus research, it has never been implemented for public use. In this article we describe the web-server RCDI/eRCDI that calculates the RCDI of a set of genes using the codon usage of a reference genome. However, the codon usage of a gene may be the product of compositional biases such as G+C or amino acids composition rather than a codon usage adaptation [9]. Therefore, an expected value of the RCDI (eRCDI) is implemented in order to test the significance of the RCDI values.

### Implementation

The RCDI/eRCDI program has been implemented in PHP as a web-server and it is freely available as one of the tools of the CAIcal server [10]. The program is also available for local use as a Perl script to calculate the RCDI and eRCDI for a great amount of sequences.

### Data input/output

The inputs to calculate the RCDI are: 1) a gene (or set of genes) in FASTA format; 2) the codon usage table of the reference genome in codon usage database [11] format and 3) the genetic code. Other input parameters are related to the calculation of the eRCDI and may be modified according to user requirements. The web-server provides 4 outputs: 1) Genes' parameters: the RCDI values, the frequencies for all codons [(CiFa/CiFh)Ni; see description below] and the %G+C of each gene; 2) Global parameters: the mean %G+C and amino acid composition, and the number of sequences used to calculate the eRCDI; 3) Statistical tests: the RCDI/eRCDI server performs a Kolmogorov-Smirnov test for normality of the eRCDI and a Chi-Square Goodness-of-Fit test to evaluate if the test sequences are homogeneous in %G+C and amino acids composition and 4) Expected RCDI: the mean RCDI value from random sequences and the eRCDI value.

### Algorithm

#### Relative Codon Deoptimization Index (RCDI)

The RCDI/eRCDI server calculates the RCDI by the equation (1), where CiFa is the relative frequency of codon i for a specific amino acid in the test sequence; CiFh is the relative frequency of codon i for a specific amino acid in the reference sequence; Ni is the number of occurrences of codon i in the test sequence; and N, the total number of codons in the test sequence. The RCDI ranges from 1 (the codon usage of the test sequence is completely optimized to the codon usage of the reference genome) to N (increases with the deoptimization of the test sequence). If the reference genome uses all 61 codons, the maximum RCDI value would be equal to the inverse of the lowest synonymous codon frequency value. E.g. for the human genome, the maximum RCDI is 17.64, in the case of a sequence made up with solely TCG codons, which have the lowest synonymous frequency (0.0567).(1)

#### Expected Relative Codon Deoptimization Index (eRCDI)

Since the codon usage of genes depends on their G+C content, it is essential to discern whether codon usage differences are statistically significant and arise from differences in codon preferences or whether they are merely artifacts that arise from internal biases in the G+C composition and/or amino acid composition of the query sequences. For this purpose, the estimation of expected values can be useful [9]. An expected value to test codon adaptation was first described for the Codon Adaptation Index (CAI) [9]. This expected value is calculated from N random sequences with a similar %G+C and amino acids composition of the query sequences. Random sequences are generated either by Poisson or Markov methods [9]. Using the same principle, an eRCDI can be calculated from the RCDI of each of these random sequences. Therefore, the eRCDI value corresponds to the upper-tolerance limit, based on the mean and standard deviation, and the level of confidence and coverage introduced by the user. The eRCDI estimated by our server provides a threshold value to estimate if a gene is deoptimized and whether this deoptimization is the product of compositional biases (%G+C and/or amino acid composition).

### Interpretation of the results

RCDI values, based on the Mueller et al. (2006) article [1], may allow for an estimation of the translation rate of viral genes in particular, and the whole genome in general. The higher the similarity between viral and host genes (close to an RCDI value of 1), the higher the translation rate is [1]. The first and most straightforward application is to improve expression of proteins in heterologous expression systems, whether increasing the translation rate or manipulating it to obtain proper folding by choosing a sequence with RCDI close to that of the viral natural host. Furthermore, from a genetic point of view, estimation of RCDI may provide insight into the degree of coevolution between hosts and viruses. A low RCDI might indicate high adaptation to a host. Moreover, a high RCDI might also indicate that some genes are expressed in latency phases or even that the virus might present a low replication rate.

### RCDI/eRCDI complements information from other indices

**Figure 1:**
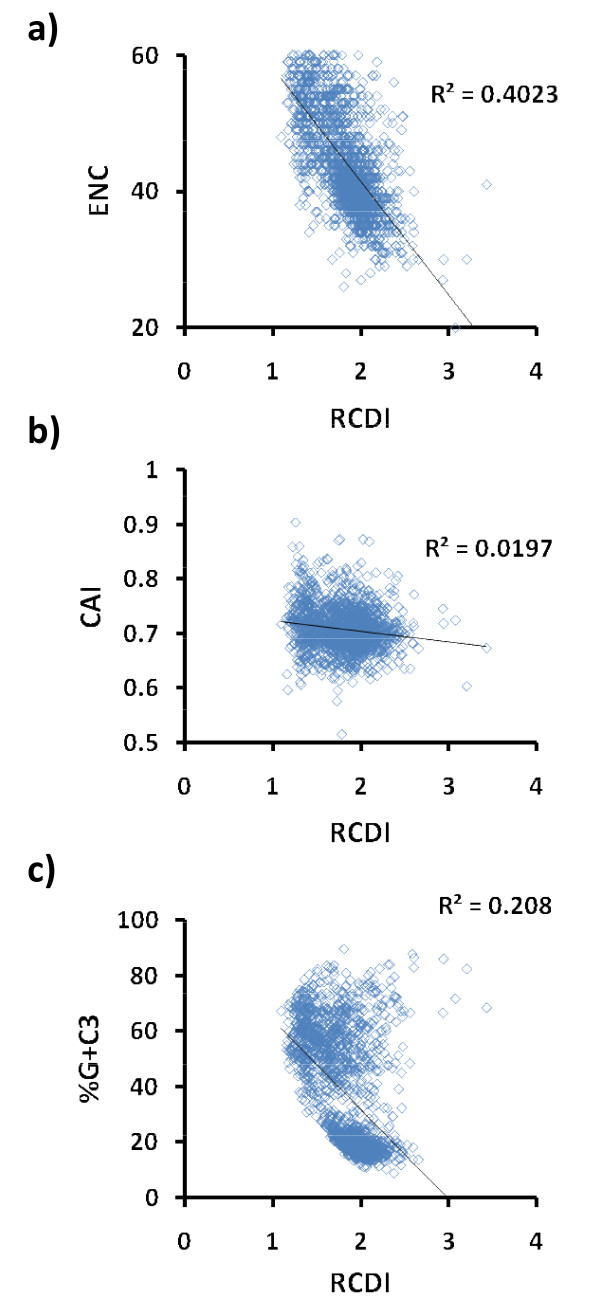
**Relative Codon Deomptimization Index (RCDI), Codon Adaptation Index (CAI), Effective Number of Codons (ENC) and G+C percentage at the third position of the codon (%G+C_3_) of 2500 viral sequences**. a) Correlation of RCDI and ENC. b) Correlation of RCDI and CAI. c) Correlation of RCDI and %G+C_3_.

The information gained from the RCDI may be complementary to that of other common indices like CAI [12] or the Effective Number of Codons (ENC) [13]. Figure 1 shows results of RCDI, CAI, ENC and G+C percentage at the third position of the codon (%G+C_3_) of 2500 viral sequences (chosen randomly from the NCBI genome resources http://www.ncbi.nlm.nih.gov/sites/genome). CAI and RCDI were calculated using the mean codon usage of the human genome as a reference set. As one may expect, there is a certain correlation (R^2 ^= 0.4023) between the ENC and the RCDI (Figure 1a). However, the ENC is a measure of how biased a gene is from an equal use of synonymous codons and it is independent of a reference genome, whereas the RCDI is genome dependent. The indices CAI and RCDI, that are both genome dependent, have a low correlation (R^2 ^= 0.0197), reinforcing the idea that these indices may be complementary (Figure 1b). CAI is a measure of codon usage adaptation to the most used synonymous codons of a reference genome [14] and is commonly used to predict highly expressed genes [15]. On the other hand, the RCDI is used to assess if the codon usage of a gene is similar to the codon usage of a reference genome. In addition figure 1c shows that RCDI values and %G+C_3 _are linearly independent.

Both indices, CAI and ENC can be associated with an expected value, the expected CAI by generation of random sequences [9] and an estimated ENC by a formula that uses the %G+C at the third position of the codon [13]. In this article we introduce the eRCDI as a measure to assess the significance of the RCDI value. Thus, based on the RCDI and eRCDI, it is possible to estimate if a gene is deoptimized and if this deoptimization is the product of compositional biases (%G+C and/or amino acid composition).

## Conclusions

The RCDI is a useful index to assess how similar the codon usage of a given gene is to the codon usage of a reference genome and to test deoptimization levels in viral genomes. However, until now, it has never been, implemented for public use. Therefore, the RCDI/eRCDI server will be a useful tool for genome analysis. For experimental virology, it will allow efficient gene design for heterologous protein expression. At a genetic level, it may help to establish and understand host-virus phylogenetic relationships as well as infer the potential host range of a virus and its replication strategy.

## Availability and requirements

• Project name: RCDI/eRCDI

• Project home page: http://genomes.urv.cat/CAIcal/RCDI

• Operating system(s): Platform independent

• Web-browser independent

• Programming language: PHP (local version in Perl and tcl/tk)

• Other requirements: local version requires perl and tcl/tk installed.

• License: no restrictions for academic and non-commercial use.

## List of abbreviations

RCDI: Relative Codon Deoptimization Index; eRCDI: expected RCDI; CAI: Codon Adaptation Index; ENC: Effective Number of Codons; %G+C_3_: G+C percentage at the third position of the codon.

## Competing interests

The authors declare that they have no competing interests.

## Authors' contributions

PP: designed the server, made the programming task and wrote the manuscript. LA: tested the server and helped to write the manuscript SG-V: helped to write the manuscript. All authors read and approved the final manuscript.
